# Should patients with abnormal liver function tests in primary care be tested for chronic viral hepatitis: cost minimisation analysis based on a comprehensively tested cohort

**DOI:** 10.1186/1471-2296-12-9

**Published:** 2011-03-03

**Authors:** David T Arnold, Louise M Bentham, Ruth P Jacob, Richard J Lilford, Alan J Girling

**Affiliations:** 1 School of Medicine, Cardiff University, Cardiff, UK; 2 School of Health and Population Sciences, University of Birmingham, Birmingham, UK; 3 NIHR Research Design Service for the East Midlands, University of Nottingham, Nottingham, UK

## Abstract

**Background:**

Liver function tests (LFTs) are ordered in large numbers in primary care, and the Birmingham and Lambeth Liver Evaluation Testing Strategies (BALLETS) study was set up to assess their usefulness in patients with no pre-existing or self-evident liver disease. All patients were tested for chronic viral hepatitis thereby providing an opportunity to compare various strategies for detection of this serious treatable disease.

**Methods:**

This study uses data from the BALLETS cohort to compare various testing strategies for viral hepatitis in patients who had received an abnormal LFT result. The aim was to inform a strategy for identification of patients with chronic viral hepatitis. We used a cost-minimisation analysis to define a base case and then calculated the incremental cost per case detected to inform a strategy that could guide testing for chronic viral hepatitis.

**Results:**

Of the 1,236 study patients with an abnormal LFT, 13 had chronic viral hepatitis (nine hepatitis B and four hepatitis C). The strategy advocated by the current guidelines (repeating the LFT with a view to testing for specific disease if it remained abnormal) was less efficient (more expensive per case detected) than a simple policy of testing all patients for viral hepatitis without repeating LFTs. A more selective strategy of viral testing all patients for viral hepatitis if they were born in countries where viral hepatitis was prevalent provided high efficiency with little loss of sensitivity. A notably high alanine aminotransferase (ALT) level (greater than twice the upper limit of normal) on the initial ALT test had high predictive value, but was insensitive, missing half the cases of viral infection.

**Conclusions:**

Based on this analysis and on widely accepted clinical principles, a "fast and frugal" heuristic was produced to guide general practitioners with respect to diagnosing cases of viral hepatitis in asymptomatic patients with abnormal LFTs. It recommends testing all patients where a clear clinical indication of infection is present (e.g. evidence of intravenous drug use), followed by testing all patients who originated from countries where viral hepatitis is prevalent, and finally testing those who have a notably raised ALT level (more than twice the upper limit of normal). Patients not picked up by this efficient algorithm had a risk of chronic viral hepatitis that is lower than the general population.

## Background

### Liver Function Tests are ordered in large numbers in primary care

Liver function tests (LFTs) are comprised of a panel of five to eight analytes that are processed inexpensively in large batches. LFTs are one of the most commonly performed "blood tests" in primary care, such that in 2003 the laboratory at University Hospital Birmingham received 67,182 requests for LFTs from 83 General Practitioner (GP) practices, serving a population of 300,000 (Cramb R; Chemical Pathology Specialist).

### Enigmatic responses to abnormal LFTs in primary care settings

An abnormal LFT may signify a serious disease that can be identified only through further testing. These conditions include liver diseases, such as primary biliary cirrhosis (PBC), diseases of other organs such as Paget's disease of bone, and multi-organ diseases such as haemochromatosis. However, the majority of people with an abnormal LFT in primary care settings will not have any such previously undetected disease. They will have either no disease at all, or will be manifesting the effects of alcohol abuse or obesity. The doctor is likely to be aware, or at least suspicious, of these behaviours when ordering LFTs, but this does not exclude the presence of other diseases that may aggravate liver damage. There is thus a real question about which specific further tests, if any, a GP should order when an abnormal LFT result is obtained in a patient with non-specific symptoms, or as a result of routine testing. In some cases there may be a clear indication for further tests. For example, if the patient has a family history of haemochromatosis then their iron saturation should be measured. In some cases the pattern of LFT abnormality may suggest a diagnosis - for example, an isolated raised unconjugated bilirubin suggests Gilbert's disease, while a high blood level of alkaline phosphatase (ALP) is indicative of PBC. In most cases however, no unambiguous clinical indication for follow-on testing exists. The literature deals mostly with the pattern of abnormality given a diagnosis, rather than the probability of the various diagnoses given a pattern of abnormal LFTs. It is therefore not surprising that guidelines for GPs [1-5] confronted by an abnormal LFT in patients with non-specific symptoms or detected fortuitously are inconsistent, or that the way GPs respond has been found to be eclectic [6]. A point on which guidelines do agree is that the LFT panel should be repeated following an abnormal result.

### Criteria for selection of a topic for decision analysis

If there is any particular previously unrecognised disease that a patient would wish to have excluded by further testing, then it will have the following features:

1. it is a serious disease;

2. it is treatable in the prodromal phase;

3. failure to identify the condition can lead to permanent damage;

4. it can be diagnosed with a high specificity by a familiar and inexpensive test;

5. it is among the more prevalent of the serious diseases;

6. it is not a condition such as alcohol misuse or obesity, which can be diagnosed from history and examination.

### Viral hepatitis

We discern that chronic viral hepatitis is the prime candidate based on the above criteria. It is a massive problem worldwide [7-9] and Table 1 shows that it is the most common of the specific liver diseases in the UK population after alcohol damage. Moreover, chronic viral hepatitis can be reliably confirmed or excluded by means of a relatively inexpensive blood test [10,11]. The disease has a prodromal period lasting many decades and is eminently treatable if caught early, thereby averting cirrhosis and liver cancer [12,13].

**Table 1:** Viral, genetic, and autoimmune diseases of the liver (tested for by a "liver panel"), their prevalence in the British population and diagnostic algorithms.*

Disease	Prevalence amongst adult population (%)	Blood tests done on all members of the cohort (to diagnose or screen for the disease)	Diagnostic algorithm
Chronic viral hepatitis C	0.42 [46]	Hepatitis C virus antibody (HCV Ab)	Viral marker positive.

Chronic viral hepatitis B	0.3 [47]	Hepatitis B viral markers (HBV Surface Ag)	Viral marker positive.

Metal storage disease: Iron	0.25 (prevalence of phenotype; homozygous plus complex heterozygous) [48]	Iron saturation	Genotype if iron saturation >50%.

Primary biliary cirrhosis (PBC)	0.024 [49]	Antimitochondrial Ab	Raised antibodies and raised ALP level.

Autoimmune hepatitis	0.001 [50]	Smooth Muscle Ab	Raised antibodies and raised ALT, AST or globulin exceeding twice the upper limit of normal. Confirmed by hepatologist.

Metal storage disease: Copper	<0.025 [51]	Caeruloplasmin	Low levels of caeruloplasmin.

Alpha-1 antitrypsin deficiency	<0.025 [52]	Alpha-1 antitrypsin	Low Alpha-1antitrypsin levels followed by phenotype testing.

* Method by which the diagnosis was made.

The purpose of the decision analysis described here is to inform the selection of an efficient strategy for the diagnosis of chronic viral hepatitis. Such a strategy should optimise the trade-off between detection rate and cost.

## Methods

### The BALLETS study

The objective of the BALLETS study was to correlate the pattern of abnormal LFTs in primary care with the 'final' diagnosis. This was accomplished by fully investigating a cohort of patients with abnormal LFTs and no known liver disease by means of a panel of tests for the specific viral, auto-immune and genetic diseases shown in Table 1. This comprehensive investigation by means of the standard "liver panel" acts like a concertina, bringing forward the diagnosis of conditions, such as viral hepatitis and PBC, that would take years or decades to manifest if followed-up in the usual way. The BALLETS study was funded by the UK National Institute for Health Research through its Health Technology Assessment (HTA) programme. The full protocol is available online [14].

Patients were recruited from eight Birmingham and three Lambeth practices from November 2005 to November 2008. The cohort was formed from patients with at least one abnormal analyte (from a panel of eight - alanine aminotransferase (ALT), aspartate aminotransferase (AST), ALP, bilirubin, gamma-glutamyltransferase (GGT), globulin (derived), total protein, and albumin) and no known liver disease. The biochemical measurements were carried out in three Clinical Pathology Accreditation UK (CPA UK) laboratories (conforming to International Quality Control Standards) in Birmingham and Lambeth. Laboratory specific ranges (incorporating age and sex adjustments for ALT, ALP and GGT) were used to define abnormality. Eligible patients from the participating GP practices were invited to attend special study clinics where the following information was obtained:

· Clinical data, including: country of birth, alcohol history, Body Mass Index (BMI), drug and medicine use, and reason for LFT testing.

· A repeat LFT.

· Blood tests for specific diseases (Table 1). Investigations for viral hepatitis tests included hepatitis B viral markers (HBV Surface Ag) and hepatitis C virus antibody (HCV Ab) [10,11].

· Ultrasound of upper abdomen.

Patients were followed up for two years, at which point LFTs were repeated along with a further ultrasound of the upper abdomen (results of the follow-on study will be reported at a later date).

### Testing strategies

A simple decision tree was constructed in Microsoft Excel (Microsoft Corp., Redmond Washington) to enable costs per case detected to be calculated for seven strategies [15]. The strategies were developed in consultation with a GP and hepatologist (P. Gill and J. Neuberger respectively) who were aware of the relevant literature and guidelines.

The root decision (or starting point) of the tree is the discovery of an abnormal LFT in primary care where the patient does not have known or self-evident liver disease. From the root node we identified seven decisions that may be considered by a GP under such a scenario:

■ Strategy A: repeat the LFT panel and then perform a specific test for viral hepatitis if an abnormality is still present on re-testing. This could be considered the intuitive response by a GP on receiving an abnormal LFT in a patient without the indictors of a specific disease, and is the strategy recommended in the literature [1-5].

■ Strategy B: perform a viral test in all patients with an abnormal ALT. The rationale for this strategy is that ALT is the most specific indicator of viral hepatitis [4], and has been recommended as the testing criterion by other authors [16-18].

■ Strategy C: select ALT as the trigger for viral testing, but nominate a higher threshold at twice the upper limit of normal, as recommended by Jamali [19]. This is also the threshold for instigating viral therapy for HBV in certain treatment guidelines [20-22].

■ Strategy D: perform a test for viral infection in all patients who originate from a country with an intermediate or high prevalence of viral hepatitis according to World Health Organisation (WHO) criteria [23-25]. Screening has been shown to be cost-effective for people who were born in prevalent countries and it is likely that testing would be more cost-effective still in a population with abnormal LFTs [21,26].

■ Strategy E: combine the two previous strategies by testing those who have an ALT exceeding twice the upper limit of normal *and *who also originate from a prevalent country.

■ Strategy F: test all patients from prevalent countries as well as those with an ALT exceeding twice the upper-limit of normal.

■ Strategy G: test all patients for viral hepatitis irrespective of the type or extent of abnormal LFT results.

There is also an option to take no action with respect to viral hepatitis, and while this may be a sound decision in some cases, for example when a LFT is ordered in the hope that a positive result will prompt a reduction in alcohol intake, this was not considered here.

In this study the hepatitis status for all patients was known. Moreover most had an ALT test result and the results of a repeat LFT panel. Thus it was possible to evaluate the performance of each of the above strategies.

### Populating the decision tree with probabilities/statistical model

All 1,236 patients were used in the evaluation of strategy G, but for all other strategies the effective sample size was reduced because of missing data in some of the patient records. Estimates of the proportion of patients undergoing viral tests, and the proportion of actual cases detected (sensitivity) were obtained using the sample of patients available for evaluating each strategy. The positive predictive value of a strategy was defined as the proportion of hepatitis cases among those selected for viral testing. Confidence limits for this quantity were calculated using Wilson's method for Binomial data [27].

### Estimation of costs

The direct costs incurred at the time of the test were the laboratory costs of the liver function and viral hepatitis tests (personal correspondence with Pathology Lab Manager); the GP costs for scheduling each test; and following up on results. Administrative costs were estimated by estimating the time implications for a secretary to add patients to appointment slots and a receptionist to check the patient in for an appointment (personal correspondence with MidReC: West Midlands Research Consortium. Figures correct as of February 2009). The costs are presented in pounds sterling (£) (and were correct for the year 2009). Non-health service costs (patient travel cost and lost earnings) were not measured, but are considered in the discussion.

### Analysis

The number of cases detected per 100 patients was estimated as the sensitivity of the strategy (cases detected divided by cases present) multiplied by the prevalence (per 100 patients) of viral hepatitis in the whole sample of 1,236 patients. For each strategy the cost per case detected was then computed as the ratio of the cost per patient to the number of cases detected per patient. The strategy which minimised this quantity was taken as the base case. For each alternative strategy the incremental cost-effectiveness ratio (ICER) was computed, defined as the incremental cost per additional case detected compared to the base case. The analysis is deterministic and does not consider the impact of sampling variability. The results of these analyses were compared with published results of cost-effectiveness analysis of screening for chronic viral hepatitis, bearing in mind likely differences between a screening and a diagnostic population. We used this analysis to develop a "fast and frugal" heuristic [28] which we offer to readers for their consideration.

## Results

### Patients

**Figure 1:**
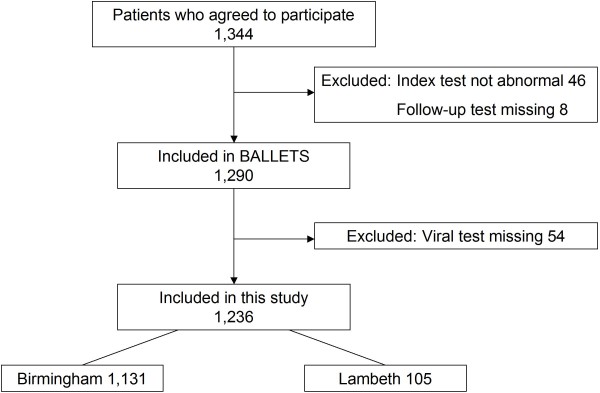
**Flow diagram of exclusions and inclusions in the study**.

One thousand, three hundred and forty-four patients consented to the study. Fifty-four were excluded because they did not match the entry criteria in the protocol, along with a further 54 where data on at least one viral hepatitis test was missing (Figure 1). This left 1,236 patients for this study. One hundred and five of these patients were from Lambeth and 1,131 were from Birmingham. The median interval between index and repeat testing was 31 days (inter-quartile range 19-52 days).

### Chronic viral hepatitis cases

Thirteen of the 1,236 patients where the test result was available had chronic viral hepatitis - nine cases of hepatitis B and four cases of hepatitis C. This gives an estimate of 1.1% (95% CI: 0.6%-1.8%) for the prevalence rate in a primary care population with abnormal LFTs; only slightly more than the baseline prevalence in the general population (0.7%). The demographic breakdown of patients with and without viral hepatitis is shown in Table 2.

**Table 2:** Demographic features of patients with and without viral hepatitis

	Total	Viral Hepatitis	Not Viral Hepatitis
**N**	1236	13	1223

**Age**			

Mean (SD)	57.7 (15.2)	54.0 (15.9)	57.7 (15.2)

**Sex**			

Male	693 (56.1%)	9 (69.2%)	684 (55.9%)

Female	543 (43.9%)	4 (30.8%)	539 (44.1%)

**Ethnic group**			

White	1023 (82.8%)	3 (23.1%)	1020 (83.4%)

Asian	88 (7.1%)	5 (38.5%)	83 (6.8%)

Black	53 (4.3%)	3 (23.1%)	50 (4.1%)

Other	38 (3.1%)	2 (15.4%)	36 (2.9%)

Missing	34 (2.8%)	-	34 (2.8%)
**Reason**			

Abdominal signs/symptoms	69 (5.6%)	1 (7.7%)	68 (5.6%)

Non-abdominal signs/symptoms	302 (24.4%)	6 (46.2%)	296 (24.2%)

Diagnosis - Alcohol abuse	17 (1.4%)	0 (0.0%)	17 (1.4%)

Review - CVD	50 (4.0%)	0 (0.0%)	50 (4.1%)

Review - Cholesterol	53 (4.3%)	0 (0.0%)	53 (4.3%)

Review - Hypertension	147 (11.9%)	2 (15.4%)	145 (11.9%)

Review - Diabetes	216 (17.5%)	2 (15.4%)	214 (17.5%)

Review - Medication	92 (7.5%)	0 (0.0%)	92 (7.4%)

Medical - Review other	290 (23.5%)	2 (15.4%)	288 (23.5%)

CVD: Cardiovascular Disease

The breakdown LFT results in the infected cases is given in Table 3. In *10 *of these *13 *cases, more than one analyte was abnormal. In *eight *cases the ALT was abnormal and was notably raised in *six *of those (above twice the upper limit of normal). In one case (perhaps detected by serendipity) only protein levels were abnormal and all the enzyme tests (ALT, AST, GGT and ALP) were normal. Eleven of the 13 patients with chronic viral hepatitis had an abnormality on the repeat LFT. There were two other cases where there were missing data among the repeat LFT panels. Of the 1,113 patients with no viral hepatitis who underwent a complete LFT panel, 169 (15%) reverted to normal.

**Table 3:** Results of initial LFT for viral hepatitis cases using laboratory-specific criteria for abnormality

	Case No.	ALT	AST	Bilirubin	ALP	GGT	Albumin	Globulin	Total Protein	Repeat LFT	Country of Origin (prevalence of viral hepatitis)
HBV	1	High*	High	Normal	Normal	High	Normal	Normal	High	Abnormal	Kenya (High)

	2	Normal	Normal	High	Normal	Normal	High	Low	Normal	Abnormal	UK (Low)

	3	High	Normal	Normal	Normal	High	Normal	Normal	Normal	Abnormal	Pakistan (High)

	4	High*	High	High	Normal	High	Normal	Normal	High	Abnormal	India (High)

	5	High*	High	Normal	Normal	Normal	Normal	Normal	Normal	Abnormal	Malaysia (High)

	6	High*	No result	No result	No result	Normal	Normal	Normal	Normal	Abnormal	UK (Low)

	7	Normal	Normal	Normal	Normal	Normal	Normal	High	High	Abnormal	Kenya (High)

	8	No result	High	Normal	High	No result	Normal	No result	No result	Abnormal	Iraq (High)

	9	Normal	No result	High	Normal	No result	Normal	No result	No result	Incomplete**	Malta (High)

HCV	1	High	Normal	Normal	Normal	High	Normal	Normal	Normal	Incomplete	Pakistan (High)

	2	High*	High	Normal	Normal	Normal	Normal	Normal	High	Abnormal	Hong Kong (High)

	3	Normal	No result	Normal	Normal	High	Normal	No result	No result	Abnormal	Jamaica (High)

	4	High*	High	Normal	Normal	Normal	Normal	Normal	Normal	Abnormal	Somalia (High)

* Denotes where ALT values were greater than twice the upper limit of normal.

** Repeat test available for AST and GGT only, both of which were normal.

ALT: Alanine aminotransferase;

AST: Aspartate aminotransferase;

ALP: Alkaline phosphatase;

GGT: Gamma-glutamyltransferase;

HBV: Hepatitis B;

HCV: Hepatitis C.

The country of origin was recorded in 1,208 of the 1,236 study cases, and of these, 170 people were born in a country with an intermediate or high prevalence of viral hepatitis (based on WHO definitions of prevalence [23-25]) and 1,038 were from low-risk countries. The high-risk group contained 11 of the 13 cases (85%) of viral hepatitis. None of the 13 cases admitted to use of intravenous drugs at any time.

As expected from the literature, ALT or AST levels when abnormal tended to be more extreme for cases with viral hepatitis than for cases that did not have this disease (Table 4).

**Table 4:** Comparison of ALT and AST results in HBV or HCV cases versus non-hepatitis cases

	Upper limit	HBV or HCV			Non-hepatitis		
**Analyte**		N	Mean	Median	N	Mean	Median

ALT	41	8	98.0	89.5	426	65.4	56.0

AST	43	6	94.5	69.5	254	64.5	53.5

Only cases where analyte is abnormal are included in this table.

ALT: Alanine aminotransferase;

AST: Aspartate aminotransferase;

HBV: Hepatitis B;

HCV: Hepatitis C.

### Diagnostic performance

The sensitivity and positive predictive value of each detection strategy are given in Table 5. It can be seen that the recommended strategy (A), of repeating the LFT and then doing a viral test if an abnormality persists, is highly sensitive. However, the predictive value is low (1.15%). Strategy D, simply carrying out a viral test if the patient originates from a high or intermediate risk country, detects 85% of cases and has a much higher predictive value of 6.47% than the strategy of repeating the LFT test. The strategy of ordering an LFT if the ALT is raised (B), is neither particularly sensitive (67%), nor does it have a high predictive value (1.91%). The more selective strategy of testing if the index ALT was over twice the upper limit of normal (C) has a higher predictive value, but is less sensitive. The best features of C and D are combined in the hybrid strategy F which achieves high sensitivity (92%) and worthwhile predictive value (5.12%).

**Table 5:** Yield, sensitivity and Positive Predictive Values (PPV) of different detection strategies

Strategy for viral testing	No. of patients*	Hepatitis cases*	Viral tests	Cases detected	Sensitivity (%)	PPV (%) 95% Confidence Limits
A. If repeat LFT panel is abnormal	1124	11	955	11	100	1.15 (0.64-2.05)

B. If ALT abnormal on primary test	1064	12	418	8	67	1.91 (0.97-3.73)

C. If ALT > 2 upper limit of normal on primary test	1064	12	77	6	50	7.79 (3.62-15.98)

D. If patient born in a country of intermediate to high viral hepatitis prevalence.	1208	13	170	11	85	6.47 (3.65-11.21)

E. If patient born in a country of intermediate to high viral hepatitis prevalence *and *ALT > 2 upper limit of normal on primary test.	1041	12	16	5	42	31.25 (14.16-55.60)

F. If patient born in a country of intermediate to high viral hepatitis prevalence, *or *ALT > 2 upper limit of normal on primary test.	1041	12	215	11	92	5.12 (2.88-8.93)

G. Test all cases	1236	13	1236	13	100	1.05 (0.62-1.79)

Testing patients for viral infection on the basis of country of origin is more sensitive and has much higher positive predictive value.

* The sample of patients available to evaluate each strategy varies because of patterns of missing data, as follows:

**A **requires a complete panel of follow-up LFTs, the missing data in the two cases that were not abnormal might have led to an exagerated estimate of sensitivity;

**B **and **C **both require an initial ALT test;

**D **requires information on country of birth;

**E **and **F **require an initial ALT together with country of birth.

**All **evaluations require results of viral tests for both Hepatitis B and Hepatitis C.

ALT: Alanine aminotransferase;

LFT: Liver function test.

### Costs and Cost Minimisation Analysis

**Figure 2:**
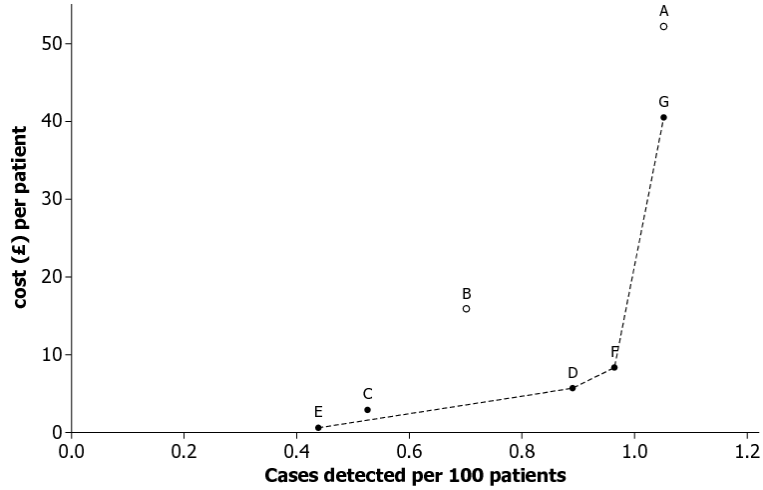
**Cost per detected case for seven testing strategies**. The number of detected cases per patient is estimated as (Sensitivity of strategy) × 1.05% where the latter figure is the viral hepatitis prevalence observed in the complete sample of 1,236 patients. The number used differs slightly from the actual number of cases detected per patient in table 5 because of variation in the prevalence of the condition across the samples in which each strategy was tested. The current approach achieves a more consistent comparison of strategies within our data-set; for example, it ensures that the estimate of detected cases per patient for a strategy with 100% sensitivity will always be at least as great as that of any other strategy.

The cost of the laboratory tests and the practice costs are given in Table 6. The average cost per case detected and the incremental costs of detecting each additional case are shown in Table 7. Strategy E (viral test if patient born in an intermediate/high-risk country *and *ALT is greater than twice the upper limit of normal) provides the lowest cost per case detected. This strategy was therefore designated as the base case for the calculation of the ICERs. Strategy A, the intuitive and widely advocated practice of repeating LFTs, turns out to be the most expensive per case detected. It is dominated by strategy G, where all patients have a viral test. Similarly strategy B (viral test if the index ALT is abnormal) is dominated by strategy D (perform viral test if patient was born in an intermediate or high risk country). Strategy C (viral test if the ALT is greater than two times the upper limit of normal) can be eliminated by an extended dominance principle. If strategy C is preferred to E, this can only be because the extra cases detected by C are deemed worth the extra cost. However, strategy D finds yet more cases than C at lower incremental cost. Therefore either E or D are preferable to C. The cost-effectiveness of the remaining admissible strategies can be visualised from Figure 2. The dotted lines join strategies that cannot be eliminated by dominance principles. The absence of any explicit penalty for missing cases of viral hepatitis in this analysis implies that the costs of E, D and F are under-estimated with respect to G. However, F must be regarded as highly competitive with G - it picks up almost as many cases and has very high efficiency in terms of cost per case detected.

**Table 6:** Cost estimates for resources used

Cost categories	Resources
GP consultation cost to check LFT results	£12.86 *

Receptionist to check patient in for appointment (2 mins)	£0.91*

Secretary time (1 min)	£0.33*

Phlebotomist time (5 mins)	£1.00*

Sample analysis: LFT	£2.69**

Sample analysis: Hepatitis B surface Ag and Hepatitis C	£25.42**

* Source: Midlands General Practice Research Consortium (MidReC, Feb 2009)

** Source: University Hospital Birmingham, NHS Foundation trust (2009)

**Table 7:** Economic analysis

Strategy	Cost per 100 patients (£)*	Cases detected per 100 patients	Cost per case detected (£)	Incremental cost per 100 patients (with base = E)	Incremental cases detected per 100 patients (base = E)	ICER
A	5222	1.05	4965	5159	0.61	Dominated**

B	1592	0.70	2270	1530	0.26	Dominated^†^

C	293	0.53	558	231	0.09	(2635)^‡^

D	570	0.89	641	508	0.45	1124

E (base)^§^	62	0.44	142	0	0.00	Base

F	837	0.96	868	775	0.53	1473

G	4052	1.05	3853	3990	0.61	6503

* Cost of Viral test = £40.52; Cost of LFT Panel = £17.79.

** Dominated by G.

† Dominated by D and F.

‡ Strategy C is eliminated by extended dominance.

§ Patient born in a country of intermediate to high viral hepatitis prevalence and ALT greater than twice upper limit of normal on primary test is the least expensive strategy and was considered as base case.

ICER: Incremental cost-effectiveness ratio.

LFT: Liver function test.

## Discussion

### Summary of main findings

The BALLETS study is the first GP based study where the entire cohort was comprehensively tested for additional diseases (such as viral hepatitis) after an abnormal LFT, using the full analyte panel and normal reference ranges. We have shown that an abnormal LFT alone does not select out a population where the prevalence rate approaches a threshold which would justify viral screening. We have assessed the validity of the various strategies a GP could adopt, at least as far as viral hepatitis is concerned, when faced with an abnormal LFT of uncertain provenance. The intuitive response for a GP in such a situation would be to repeat the LFT, an approach advocated by current literature. This study shows that this may not be the optimal policy. This strategy is the most expensive, even more so than viral testing all patients, as the costs incurred include repeating the LFT as well as viral testing the majority. The study also shows that if ALT is notably raised (greater than twice the upper limit of normal), then the probability of chronic viral hepatitis is high (nearly 8%), but sensitivity is low. The strategy of testing all people from prevalent countries is the second most efficient, in terms of cost per case detected, and detects almost twice as many cases as the most efficient strategy - testing for viral infection when two conditions (birth in a prevalent country and an ALT greater than twice the upper limit of normal) are satisfied. The relative financial disadvantages of the strategy of repeating the LFT would be even greater if patient costs were included, as the extra visit would have to be factored in.

### Previous literature on LFTs and liver diseases

We conducted a literature review using the search strategy shown in Table 8, with the aim of retrieving papers that studied a cohort of patients with abnormal LFT results to provide evidence on the probability of various liver diseases (including chronic viral hepatitis) *given *abnormal test results. Any such studies would enable the precision of our observations to be strengthened. The search strategy returned 1,448 papers, including a previous review by Dufour et al. (2000) [29].

**Table 8:** Search strategy for studies looking prospectively at patients who have received an abnormal LFT*

Liver Function Test Search Strings (*limited using the subheadings; blood, analysis and metabolism*)	Hepatitis Search Strings
Liver Function Test	Liver Diseases (diagnosis)

Transaminases	Liver Diseases (epidemiology)

Alanine Aminotransferases	Liver Diseases (enzymology)

Aspartate Aminotransferases	Liver Diseases (virology)

Alkaline Phosphatase	Liver Diseases

Gamma-Glutamyltransferases	

*With Limits added (Humans and Pub date post 1980)*	*With Limits added (Humans and Pub date post 1980)*

**Papers returned = 35070**	**Papers returned = 8526**

When strategies combined using term AND = **1448 papers **whose abstracts were read	

*Based on Medline and limited to human studies after 1980.

Only eight studies matched our requirement of following up patients with an abnormal LFT result. Two additional articles were selected from the references of relevant studies. As a result, to the best of our knowledge, there are only ten studies where a cohort of asymptomatic patients with abnormal LFTs were followed up (Table 9). However, one article was written in Korean (only the abstract was translated), so was excluded from our analysis.

**Table 9:** Studies that have followed up patients from the general population after abnormal LFT

Author and Country	Date	Type of Study and population studied	Analytes Used	Patients enrolled	Patients with abnormal LFTs (%)	Prevalence of viral hepatitis in patients with abnormal LFTs	Notes
McLernon DJ et al. [31] Scotland	2009	Record Linkage; laboratory database of GP tests, hospital admissions and death certificates.	Bilirubin, Albumin, ALP, GGT, ALT, AST (transaminases sometimes combined). GP selected	95,977	20,827 (21.7%)	2.2%	Median follow up of 3.7 years. Risk of under ascertainment.

Pendino GM et al. [35] Italy	2005	Prospective Cohort Study; general population.	AST, ALT, GGT.	1,645	319 (19.4%)	17.9%	High baseline rate of viral hepatitis; 5.6%.

Kim HC et al. [30] Korea	2004	Record Linkage: insurance data and death certificates.	AST, ALT.	142,055	11,193 (7.9%)	N/A	Outcome was liver disease mortality.

Yano E et al. [16] Japan	2001	Prospective Cohort Study; "healthy" office workers.	AST, ALT, GGT.	1,973	358 (18.1%)	2.7%	Assumed that all liver cancer and cirrhosis was a result of viral hepatitis.

Daniel S et al. [36] USA	1999	Prospective Cohort Study; primary care population.	ALT, AST raised 50% above normal on at least two occasions across a six month period.	1,124	1,124 (100%)	N/A	Marker was negative patients only, so infected patients excluded from analysis.

Mathiesen UL et al. [37] Sweden	1999	Prospective Cohort Study; primary care population.	AST, ALT raised for at least 6 months. (ALP had to be normal).	150	150 (100%)	15.3%	

Whitehead MW et al. [39] UK	1999	Prospective Cohort Study; primary care population.	AST markedly raised (10 times (>400 U/l) above the upper limit of normal.)	137	137 (100%)	2.2%	

Bellentani S et al. [34] Italy	1994	Prospective Cohort; general population.	AST, ALT, GGT.	6,917	1,473 (21.3%)	2.4%	

Hultcrantz R et al. [38] Scandinavia	1986	Prospective Cohort Study; primary care population.	AST, ALT moderately raised for at least 6 months.(ALP had to be below twice the upper limit of normal).	149	149 (100%)	2.7%	

We also identified a relevant study by Kim and colleagues. This study prospectively followed a group of "healthy" Korean factory workers taking measurements of ALT, AST and GGT on at least two separate occasions. The full article was in Korean so we only had access to the abstract [53].

ALT: Alanine aminotransferase;

AST: Aspartate aminotransferase;

ALP: Alkaline phosphatase;

GGT: Gamma-glutamyltransferase;

HBV: Hepatitis B;

HCV: Hepatitis C;

LFT: Liver function test.

Two of the remaining nine English language papers described record linkage studies. One such study was based on the Korean insurance database that was linked with death certificates [30]. This study reported that increased ALT, even within the upper end of the normal range, was associated with eventual death from liver disease. A study carried out in Scotland linked general practice and hospital databases [31,32]. However, this was a retrospective study so a full liver screen was not conducted and follow-up was for a median of four years only, whereas many diseases, including chronic viral hepatitis, have much longer prodromal periods [33].

The other seven studies were prospective cohort studies, based on testing asymptomatic members of the general population. The famous Dionysos study based on three analytes from the LFT analyte panel [34] is included among these. In this study an impressive 6,917 citizens from two communities in northern Italy were screened. Although they tested all those who had an abnormal LFT (n = 1473) for viral hepatitis, for which they found a prevalence rate of 2.4%, they did not describe the pattern of LFT results in infected patients. Another Italian study by Pendino and colleagues (2005) screened 1,645 inhabitants from a town in southern Italy, with both a LFT (ALT, AST and GGT) and viral screen [35]. The prevalence of viral hepatitis is much higher in this region because of a significant immigrant population, and they performed a more extensive analysis on the impact of viral hepatitis on LFTs. Of the 319 (19.4%) individuals who received an abnormal LFT, nearly 18% were infected with viral hepatitis. However, the LFT missed 34 (40%) of the 92 cases of viral hepatitis present in the community. Perhaps the most comprehensive prospective analysis looking at the effect of viral hepatitis on the individual analytes was carried out on a population of Japanese office workers [16]. The study used data from compulsory health checks, which included an ALT, AST and GGT panel along with certain additional tests, such as a viral screen, that were added for study purposes. They found that ALT was the most sensitive of the three analytes used, detecting nearly half the cases of viral hepatitis, whilst being abnormal in 14% of the cohort (278 abnormal results in 1,973 participants). The remaining four prospectively designed studies were carried out in general practices and were therefore closer in population terms to the BALLETS cohort. However, three of these were restricted to patients with persistently abnormal LFTs over a six month period [36-38] and one of these did not include a test for viral hepatitis. The final prospective study by Whitehead (1999) was small and based on only one analyte [39].

After this review of the literature we concluded that there has been no published study that fully investigated a cohort of patients in primary care with an abnormal LFT result (from the full LFT analyte panel).

### Strengths and limitations of the study

The main strength lies in the unique nature of the BALLETS cohort, being the only prospective study that has looked at the consequences of an abnormal LFT from a full analyte panel in primary care. The main limitation of our study relates to the rather small number of cases of chronic viral hepatitis (n = 13) and hence wide confidence limits on the results. That said the results are plausible, in the sense that they are consistent with the pathophysiology of hepatitis and in line with what was found in non-practice settings (see literature review above). They are available for meta-analysis with potential future studies.

We deliberately selected multi-cultural inner city populations in order to provide a sizable sub-group of people from countries where chronic viral hepatitis is common, as a result of infection during infancy (hepatitis B),[40] and iatrogenic infection (hepatitis C).

Our study considers only one disease type, chronic viral hepatitis, while GP decision making must take into account other diseases, such as haemochromatosis, as well as other behavioural and social motivations for testing [41,42]. That said, our conclusion that repeating the LFT "offers more than it delivers," may well apply to diseases such as PBC and haemochromatosis.

Lastly we have presented an analysis for cost minimisation and incremental cost per case detected. This is not a full cost-effectiveness or decision analysis. Donnan et al. did attempt a decision analysis [32]. However this decision analysis was intended to find the most cost-effective strategy in the short term and used a limited time horizon of one year. LFTs are often ordered to prevent poor outcome in the long term, with many serious liver diseases, viral hepatitis included, manifesting over decades. Anxiety resulting from a false positive result was included in the model while long term health gains as a result of successful case finding and treatment were not captured.

Neither our decision analysis, nor that in Donnan's HTA report [32], considered cost-effectiveness. We tackle this limitation by considering our results in the context of published cost-effectiveness analyses for screening for viral hepatitis (i.e. studies that found screening was cost-effective in populations with high prevalence rates e.g. migrants) and attempt to produce a "fast and frugal heuristic" [28] to guide practice.

### Implications for practice: a fast & frugal heuristic

The intuitively appealing practice of repeating abnormal LFTs (strategy A) gets little support from our analysis. It is the most expensive option, both in absolute terms and in terms of cost per case detected, compared to all five alternative strategies (Table 7) - including that of simply testing everyone for viral infection.

The most important question a doctor can ask a patient with abnormal LFTs is their country of origin. This holds good whether the person settles in an area of high or low ethnic mix, since infections are acquired in infancy (hepatitis B), or as a result of sub-standard medical practices, such as needle sharing (hepatitis C). Once infected, people "take their risk with them" - less people will need to be tested in a low ethnic mix area, but those from prevalent countries still need testing. The strategy of testing people from prevalent countries promises good value for money. In this study, 11 of the 13 cases originated in medium or high risk countries. Thus the prevalence of chronic hepatitis viral infection (positive predictive value) among people with an abnormal LFT who were born in a prevalent country was 6.5% (11/170, 95% CI: 3.7%-11.2%, see table 5), while the prevalence among the home born population (of all ethnic groups) was less than 0.2% (2/1038, CI: 0.05%-0.7%). Our findings support viral testing only in the former group, consistent with the threshold prevalence for both HBV and HCV, of approximately 3% at which population screening becomes cost-effective [21,43,44].

Four of the strategies, C, D, E and F, entail viral testing in a population where the rate of hepatitis exceeds the 3% threshold for which testing has proven cost-effective in screening programs (Table 5). The cost-effective threshold is probably a little lower in a diagnostic population than in a screening population (costs of inviting people to attend are lower and cases detected might be a slightly higher risk) but no other strategy yields a population with a hepatitis rate exceeding even 2%.

**Figure 3:**
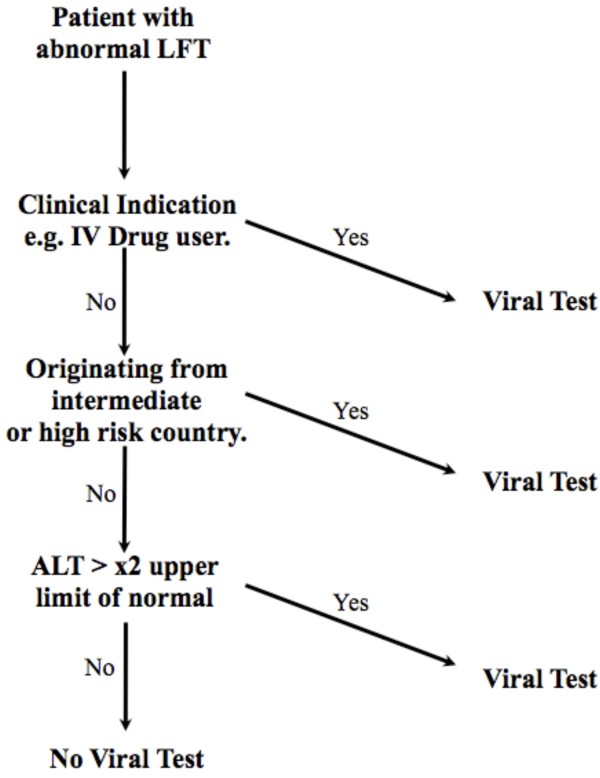
**Fast and frugal heuristic decision tree**.

Strategy D (test immigrants from prevalent countries) has a better (lower) incremental cost-effective ratio than C and detects twice as many cases as E. However, the strategy F, testing immigrants from prevalent countries *or *any people with a very high ALT, is our preferred strategy, being both sensitive and efficient. We therefore recommend the "fast and frugal" heuristic described in Figure 3. This combines strategy F with normal judgement of clinical indications. For example a patient who is an intravenous drug user, or who has recently returned from a trip abroad where they had an attack of hepatitis, would be tested notwithstanding the result of the LFTs. Otherwise we recommend testing all patients with an abnormal LFT who were born in a country of intermediate or high prevalence and all patients for whom the ALT exceeds twice the limit of normal.

The probability of chronic viral hepatitis is low even when the ALT exceeds this limit and the patient does not originate from a medium or high-risk country (1.6%). Nevertheless we advocate testing in these patients for the following reasons:

1. It is hard to ignore a level this high, and the wide confidence levels from our data suggest the need for flexibility [45].

2. The progression for undetected chronic viral hepatitis is worse for patients with ALT greater than twice the upper limit of normal, and this level has been used as a threshold for treatment in guidelines.

3. If chronic viral hepatitis is not present at this level a more in-depth search for other causes of hepato-cellular damage is indicated.

We draw the line on further viral testing after this algorithm has been followed, unless of course further clinical indicators emerge. The likelihood of a case of viral hepatitis being present following the exclusions in this algorithm is approximately 0.1% in our study. This is considerably below the UK population prevalence.

## Conclusions

This analysis indicates that the strategy of repeating LFTs in asymptomatic patients, advocated by current guidelines, is less sensitive and far more expensive than viral testing those patients born in countries where viral hepatitis is prevalent. Despite few cases of viral hepatitis the data on costs of the various strategies is strong and the results of prevalence rates within the cohort are consistent with other literature. The finding that a notably raised ALT level was also effective at identifying infected patients inspired the construction of a "fast and frugal" heuristic that might aid GPs who are faced with abnormal LFTs in asymptomatic patients, with regards to viral hepatitis. Our proposal addresses the diagnostic problem by identifying a clear high-risk population originating in prevalent countries. The residual population who are not immigrants from such countries are at low risk. However, this should not override clinical judgement. Its overall cost in other settings will depend on the relative proportions of patients in these risk-strata, but our results suggest that the cost of automatic testing of high-risk individuals will be repaid in terms of additional cases detected.

Clearly the situation might change as vaccination catches on in developing countries and needle hygiene improves. The key points to emerge are that:

1) it is more efficient to determine country of origin with a view to viral testing, than to simply repeat the LFT;

2) it is more cost-effective to test the whole LFT positive population for viral hepatitis, than to repeat the LFT with a view to viral testing if it remains positive.

## Competing interests

The authors declare that they have no competing interests.

## Authors' contributions

DTA, RPJ, RJL and AJG were involved in drafting the manuscript. DTA and RPJ were involved in the data interpretation. LMB carried out data collection and preliminary analysis. AJG performed the quantitative analysis. RJL conceived the study. All authors read and approved the final manuscript.

## Author Information

DTA - Medical student, Cardiff University

LMB - BALLETS study coordinator, University of Birmingham

RPJ - Formerly researcher for BALLETS; currently Health Economist, University of Nottingham

RJL - Professor of Clinical Epidemiology, University of Birmingham

AJG - Research fellow, University of Birmingham

## Pre-publication history

The pre-publication history for this paper can be accessed here:

http://www.biomedcentral.com/1471-2296/12/9/prepub
